# Landscapes and Human Health

**DOI:** 10.3390/ijerph14101212

**Published:** 2017-10-11

**Authors:** William C. Sullivan, Chun-Yen Chang

**Affiliations:** 1 Department of Landscape Architecture, University of Illinois at Urbana-Champaign, Urbana, IL 61801, USA; 2 Department of Horticulture and Landscape Architecture, National Taiwan University, Taipei City 10673, Taiwan; cycmail@ntu.edu.tw

As urbanization increases around the world and fewer and fewer people have easy access to completely natural places, there is a growing need to understand how the landscapes we design and inhabit impact our health and wellbeing. Over the past 30 years, we have come to understand that exposure to nature, even urban nature, impacts humans’ capacity to pay attention [1,2], recover from stressful events [3,4], and form stronger social ties among neighbors [5]. Still, a number of urgent questions remain.

This special issue of IJERPH, “Landscapes and Human Health” addresses a number of these questions. Here, you will find 19 articles: 15 reporting empirical work, 3 systematic reviews of the literature, and 1 describing a compelling new theory.

The empirical articles grapple with a range of independent variables almost all of which measure varying exposures to natural elements within a landscape: vegetation within a neighborhood, proximity to parks, participation in forest bathing, proximity to water, and contact with traditional gardens or wind farms. Some of these papers measure not only the quantity of exposure to nearby nature but also the quality of the nature experience.

The empirical articles assess the impact of these landscape features on three broad categories of outcomes: mental health, physical health, and social health. The articles assessing mental health measure some combination of quality of life, wellbeing, depression, mood states, children’s strengths and difficulties, and soft fascination. The articles assessing physical health measure some combination of cardiovascular functioning, brain functioning, birth outcomes, mobility, health behaviors, asthma, and heat-related ambulance calls. The articles assessing social health examine family dynamics or satisfaction with one’s neighborhood.

That three of the papers in this special issue are systematic reviews speaks to the volume of recent research on landscapes and human health. The Browning and Lee review examines the amount of “greenness” within a 250-m, 500-m, 1000-m or a 2000-m buffer surrounding a person’s home as a predictor of health outcomes. Browning and Lee examined 260 analyses in 47 articles and report that larger buffer sizes, up to 2000 m, better predicted physical health than smaller ones.

The Lee et al. review examines the extent to which participation in forest therapy programs were successful in decreasing depression among adults. They examined 28 articles and report evidence of the effectiveness of forest therapy programs. They conclude, however, that on the whole, the 28 studies lacked methodological rigor. They call for more rigorous study designs in future studies, assessing the long-term effects of forest therapy on depression.

The Zhang et al. review examines the extent to which natural environments convey health benefits to people with mobility impairments. They examined 27 articles and report that exposure to natural settings has positive health impacts for people who have limitations on their mobility. The health benefits spanned a range of physical, mental, and social health outcomes. Like the Lee et al. review, Zhang and his co-authors call for more methodological rigor in future studies.

This special issue also includes a compelling theory paper by Pretty, Rogerson, and Barton. Building on evidence from a range of fields, they propose a Green Mind Theory, offering a mechanism through which entire societies might address pervasive health challenges if people adopt green mind habits.

Taken together, the articles presented here contribute to the explosion of evidence demonstrating that exposure to landscapes with natural elements has pervasive, positive, prolonged impacts on human health [6]. These natural elements need not be vast in scale nor dramatically beautiful to benefit human health. Exposure to neighborhood street trees, small parks, or views of nature out a window all have salutary impacts on health. What seems more important than scale is the frequency of exposure to nature. The vast majority of the evidence demonstrates that more frequent contact with nature predicts better health outcomes.

Still, there are important questions to address. After reading the papers here, you may be struck by how much we still need to understand about the dose–response relationship between exposure to natural elements in the landscape and health outcomes. We know relatively little about the main effects of duration of exposure, frequency of exposure, and density of nature to which people are exposed on health outcomes. We know even less about the interactions among these measures of exposure.

We have growing confidence that the benefits of nature exposure are available to people in a wide variety of cultures and at various points across the human life-span. In the studies reported here, we see evidence of the health benefits of contact with nature for people in China, Korea, Taiwan, Europe, and North America. These benefits extend to children, adults, older people, mothers with young children, pregnant women, individuals suffering from cardiovascular disease, depression, anxiety, stress, asthma, and mobility impairments. 

These findings encourage us to dig deeper. What exposure pathways (e.g., visual versus tactile; direct versus through a window versus on a screen) effectively promote health? To what extent do varying forms of exposure impact mental health, physical health, social health, and health behaviors [6]? Are the health benefits of exposure to nearby nature consistent across the life span?

In spite of these questions, it is becoming increasingly clear that regular exposure to nearby nature offers hope and health to individuals and communities grappling with high levels of stress, mental fatigue, social isolation, rising rates of obesity, and sedentary behavior [7]. Yes, we need more research to understand the mechanisms through which these benefits occur. We have enough faith in the findings, however, to make it clear that cities should take the steps necessary to ensure there is nature at every doorstep.
